# PD-L2 deficiency in Alveolar macrophages drives fibrosis, apoptosis, and ferroptosis via M1 polarization in connective tissue disease-associated interstitial lung disease

**DOI:** 10.1007/s10238-026-02115-5

**Published:** 2026-03-13

**Authors:** Junhui Lu, Xiuyuan Feng, Xin Chang, Wei Cheng, Pengfei Pan, Jian Wu

**Affiliations:** 1 Department of Rheumatology, The First Affiliated Hospital of Soochow University, Suzhou, 215006 China; 2 Department of Rheumatology, The Second People’s Hospital of Huai’an, The Affiliated Huai’an Hospital of Xuzhou Medical University, Huai’an, 223000 China; 3 Department of Rheumatology, Wuxi Huishan District People’s Hospital, Wuxi, 214100 China; 4 Jiangsu Institute of Clinical Immunology & Jiangsu Key Laboratory of Clinical Immunology, The First Affiliated Hospital of Soochow University, Suzhou, 215006 China; 5 Department of Dermatology, The Affiliated Changshu Hospital of Nantong University, Suzhou, 215006 China; 6 The First Affiliated Hospital of Soochow University, 899 Pinghai Road, Gusu District, Suzhou, 215006 Jiangsu Province China

**Keywords:** Connective tissue disease-associated interstitial lung disease, PD-L2, Macrophage polarization, Fibrosis, Ferroptosis

## Abstract

Macrophages play a pivotal role in modulating immune responses in connective tissue disease-associated interstitial lung disease (CTD-ILD). The role of programmed death ligand-2 (PD-L2), an immune checkpoint molecule expressed on macrophages, in macrophage polarization in CTD-ILD remains poorly understood. Serum PD-L2 levels were measured in CTD-ILD patients, CTD-nonILD patients, and healthy controls. Alveolar macrophages (AMs) were transfected with PD-L2-targeting shRNA or control constructs, and their effects on macrophage phenotype and fibroblast fate were evaluated. M1/M2 polarization was assessed by RT-PCR, Western blotting, and flow cytometry. Human embryonic lung fibroblasts (HELFs) were co-cultured or treated with macrophage-conditioned media, and assays for cell viability, apoptosis, fibrosis, and ferroptosis were performed. A bleomycin (BLM)-induced CTD-ILD mouse model was used to evaluate the effects of PD-L2 knockout on lung fibrosis and ferroptosis markers. Serum PD-L2 levels were significantly lower in CTD-ILD patients compared with CTD-nonILD patients and healthy controls, and negatively correlated with the extent of lung fibrosis. In vitro, PD-L2 knockdown in AMs promoted M1 polarization, suppressed M2 related markers, and induced fibroblast apoptosis, fibrosis, and ferroptosis. Conditioned media from PD-L2-deficient macrophages produced similar effects. In vivo, PD-L2 knockout mice exhibited decreased numbers of CD206^+^ macrophages and regulated ferroptosis markers (ACSL4 upregulation, GPX4 and FTH1 downregulation) in lung tissues following BLM treatment. This study identifies PD-L2 as an important regulator of macrophage polarization and fibroblast responses in CTD-ILD. Our findings suggest that serum PD-L2 levels may reflect disease severity, and that restoring PD-L2 function could represent a potential therapeutic direction warranting further investigation in preclinical models.

**Supplementary Information:**

The online version contains supplementary material available at 10.1007/s10238-026-02115-5.

## Introduction

Interstitial lung disease (ILD) is a frequent and serious complication of connective tissue diseases (CTDs) [1, 2], which include systemic sclerosis (SSc), rheumatoid arthritis (RA), and polymyositis (PM) [3]. ILD involves chronic inflammation and progressive fibrosis of the pulmonary interstitium, leading to irreversible impairment in lung function [4, 5]. Importantly, CTD-associated ILD (CTD-ILD) is a major contributor to morbidity and mortality in affected patients, yet its pathogenesis remains incompletely understood [6]. Although anti-inflammatory and anti-fibrotic treatments are available, they offer limited efficacy [3], highlighting the urgent need to identify new cellular and molecular targets that can modulate disease progression.

As key components of the innate immune system, macrophages play a central role in the pathogenesis of CTD-ILD [7]. Inflammatory signals in the lung microenvironment recruit and activate macrophages, which in turn influence both the inflammatory response and fibrotic remodeling [8]. Specifically, macrophages can be activated into the classically activated phenotype (M1), which is involved in pro-inflammatory responses, and the alternatively activated phenotype (M2), which plays a key role in anti-inflammatory responses [9]. M1 macrophages typically produce pro-inflammatory mediators (e.g., IL-1β, IL-6, IL-12, and IL-23) and iNOS-dependent reactive nitrogen intermediates, contributing to epithelial injury and oxidative stress and thereby facilitating early tissue damage and remodeling [10, 11]. In contrast, persistent inflammatory signaling and chronic injury can promote M2-skewed responses [12]. M2-like macrophages are frequently enriched in fibrotic ILDs and are strongly associated with fibroblast activation and extracellular matrix deposition [12]; for example, they can produce profibrotic mediators such as TGF-β, which promotes fibroblast-to-myofibroblast differentiation [13, 14] and epithelial-mesenchymal transition [15]. In CTD-ILD, the balance and transition between these states may critically affect the severity and outcome of lung fibrosis [11]. However, the molecular mechanisms that regulate macrophage behavior in this context are still poorly defined.

Programmed death ligand-2 (PD-L2) is an immune checkpoint receptor ligand and belongs to the B7 protein family [16]. PD-L2 is primarily expressed on antigen-presenting cells, such as macrophages and dendritic cells [17]. Beyond its well-known role in regulating adaptive immunity, emerging evidence suggests that PD-L2 also participates in innate immune regulation, particularly in macrophage activation and polarization [18, 19]. Several studies have reported that PD-L2 expression is dynamically regulated during macrophage activation and is associated with M2-like polarization states in specific pathological contexts [20, 21]. For example, loss of latexin (LXN) promotes macrophage M2 polarization and PD-L2 upregulation, thereby suppressing T-cell responses and facilitating tumor growth in mouse models [20]. Similarly, PD-L2 overexpression in tumor-associated macrophages has been implicated in immunosuppressive tumor microenvironments [21]. However, these observations are based on cancer or infection-related models, and whether PD-L2 plays a comparable role in regulating macrophage phenotype and fibroblast responses in CTD-ILD has not been well defined. Given the central contribution of macrophage-fibroblast crosstalk to fibrotic progression in CTD-ILD, elucidating the functional role of PD-L2 in this context may provide new insights into disease-specific immune regulation.

In this study, we investigated the role of PD-L2 in macrophage-mediated regulation of fibrosis in CTD-ILD, with a particular focus on its effects on macrophage phenotype and macrophage-fibroblast interactions. Clarifying whether PD-L2-associated macrophage polarization is specific to CTD-ILD, as opposed to other fibrotic lung diseases such as post-COVID fibrosis, may have important implications for mechanism-guided therapeutic targeting.

## Materials and methods

### Patient data collection

The clinical data of patients with CTDs who were hospitalized in the Department of Rheumatology at the Second People’s Hospital of Huai’an between January 2024 and September 2024. Patient demographics (age and sex), specific CTD diagnoses, and laboratory test results were extracted from their electronic medical records. The healthy controls consisted of healthy individuals matched by age and sex who underwent routine physical examinations at the hospital during the same period. Patient serum samples were collected at the time of hospitalization and preserved at -80 °C until use. The study was approved by the Institutional Ethics Committee (Approval No. HEYLL2024059). All participants provided written informed consent. For all CTD patients, the inclusion criteria were: (1) newly diagnosed with CTD; and (2) treatment-naïve or had discontinued glucocorticoids and immunosuppressants for at least six months. The exclusion criteria were: (1) coexisting pulmonary infections; (2) other pulmonary diseases such as pneumoconiosis, radiation pneumonitis, sarcoidosis, or COPD; (3) significant comorbidities such as heart failure, hepatic/renal insufficiency, or pulmonary hypertension; (4) malignancies; and (5) pregnancy or lactation. For specific CTD diagnoses, patients with RA met the 2009 ACR/EULAR classification criteria. Patients with systemic lupus erythematosus (SLE) met the 2012 SLICC criteria. Patients with primary Sjögren’s syndrome (pSS) met the 2012 ACR diagnostic recommendations. Idiopathic inflammatory myopathy (IIM) cases were classified according to the 2022 ENMC criteria for dermatomyositis (DM), the 1975 Bohan and Peter criteria for DM/PM, or the 2017 ENMC criteria for immune-mediated necrotizing myopathy (IMNM). SSc was defined by the 2013 ACR/EULAR criteria. Mixed connective tissue disease (MCTD) was diagnosed according to the 1991 Kahn criteria, and undifferentiated CTD (UCTD) met the 1999 Mosca criteria.

All CTD patients were divided into two groups: those with ILD (CTD-ILD group) and those without ILD (CTD-nonILD group). For the CTD-ILD group, ILD diagnosis was based on characteristic high-resolution computed tomography (HRCT) findings, including reticular patterns, consolidation, traction bronchiectasis, honeycombing, ground-glass opacities, or nodules. HRCT scans were performed with patients in the supine position using spiral scanning at end-inspiration, from the lung apex to base. Scan parameters: tube voltage 120–130 kV, slice thickness 1.25 mm, and window settings 1400/-600 HU. The extent of ILD was semi-quantitatively assessed using the Goh scoring method. Three anatomical levels were evaluated, and the percentage of fibrotic involvement in the total lung area was estimated and scored as follows: 0: no abnormality; 1: 1–25% involvement; 2: 26–50%; 3: 51–75%; 4: >75%. The total ILD score was calculated by summing the five level-specific scores. All scans were independently reviewed and scored by two experienced radiologists, and the average of the two scores was used in the analysis.

## Cell culture

Human embryonic lung fibroblast (HELF) cell line and human alveolar macrophages (AMs) were purchased from Procell (Pricella Biotechnology, Wuhan, China). The HELF cells and AMs were cultured in Dulbecco’s modified Eagle’s medium (DMEM, Servicebio, Wuhan, China) supplemented with 25 mM HEPES, 4 mM L-glutamine, and 10% fetal bovine serum (FBS, Gibco, USA). All cells were cultured in a sterile incubator at 37 °C with 5% CO_2_.

AMs were induced into M0-phase macrophages using 10 ng/mL Phorbol-12-myristate-13-acetate (PMA, Sigma-Aldrich, USA) for 24 h. M0-phase macrophages were induced into M1-phase macrophages by treatment with 1 µg/ml LPS (Sigma-Aldrich, USA) for 4 h. M0-phase macrophages were induced into M2-phase macrophages by treatment with 50 ng/ml IL-4 (Yeasen Biotechnology, Shanghai, China) for 24 h. HELF cells were treated with 5 ng/mL TGF-β1 (Medchem-Express, USA) for 48 h to induce fibrosis.

To evaluate the effects of macrophage-derived soluble factors on fibroblasts, a Transwell co-culture system was employed. Briefly, AMs and HELF cells were seeded into 6-well Transwell plates with 0.4-µm pore inserts (Corning, USA). AMs were seeded into the upper chambers at approximately 60% confluence and subjected to shPD-L2 transfection or stimulation with 1 µg/mL LPS or 50 ng/mL IL-4 as described above. HELF cells were seeded into the lower wells and pre-treated with 5 ng/mL TGF-β1 for 24 h to induce fibrotic priming. After 48 h of HELF preconditioning, the culture medium in the lower wells was refreshed. The Transwell inserts containing AMs were then transferred into the corresponding HELF-containing wells. After 24 h of co-culture, HELF cells were harvested for downstream assays, including apoptosis, viability, fibrosis, and ferroptosis analysis.

AM-conditioned supernatants were prepared as follows. AMs were seeded into 6-well plates and treated with the indicated conditions (shCtrl, shPD-L2, LPS, or IL-4). After 48 h of incubation, the culture media were collected and centrifuged at 300 × *g* for 5 min at 4 °C to remove cellular debris. The resulting supernatants were then passed through a 0.22-µm filter to eliminate residual cells and debris and stored at − 80 °C until use. Prior to treatment, AM-conditioned supernatants were thawed, equilibrated to 37 °C, and mixed 1:1 with fresh HELF culture medium. HELF cells were incubated with the mixed media for 24 h before being harvested for subsequent analyses.

## Cell transfection

ShRD-L2 RNA, shCtrl RNA, PD-L2 overexpression plasmid vector, and the empty control plasmid vector were synthesized by GeneChem (Shanghai, China). The macrophage transfection was performed using the Lipofectamine 3000 reagent (Invitrogen, USA) according to the manufacturer’s instructions.

## RT-PCR

RNA was extracted from cells using Trizol reagent (Yeasen Biotechnology, Shanghai, China) following the manufacturer’s instructions. MultiScribe™ reverse transcriptase (Applied Biosystems, USA) was used to convert 4 µg of total RNA to cDNA. Fast SYBR^®^ Green Master Mix (Applied Biosystems) was used to amplify cDNA. RNA quantification was performed using RT-PCR (StepOnePlus, ThermoFisher, USA). The amplification protocol consisted of an initial denaturation at 95°C for 2 min, followed by 40 cycles of denaturation at 95°C for 15 s and annealing/extension at 60°C for 30 s. Melt curve analysis was performed with the following conditions: 95°C for 5 s, 65°C for 5 s, and 95°C for 30 s. Gene expression levels were normalized to GAPDH and calculated using the 2^−ΔΔCt method. The primer sequences used in this study were as follows: PD-L2, forward primer, 5’-TTCCTCCTGCTAATGTTG-3’, reverse primer, 5’-AACTGGCTGTTATTGCTC-3’; GAPDH, forward primer, 5’-GACCTGACCTGCCGTCTA-3’, reverse primer, 5’- AGGAGTGGGTGTCGCTGT-3’.

## Western blotting

Proteins were extracted from cells or lung tissues using the RIPA lysis buffer (Beyotime, Shanghai, China). The total protein concentration was measured using the BCA protein assay kit (HyClone-Pierce, Guangzhou, China). A total of 20 µg of protein extracts were loaded onto SDS-10% polyacrylamide gels, separated using electrophoresis, and transferred to polyvinylidene fluoride (PVDF) membranes. The membranes were blocked with 5% skim milk for 2 h, washed three times with TBST, and incubated with primary antibodies overnight at 4 °C. Subsequently, the membranes were washed three times with TBST and incubated with HRP-labeled secondary antibodies (1:3000) for 1 h at room temperature. The membranes were then washed again and imaged using an ECL detection system (P0018M, Beyotime, China). The antibodies used in this study are listed in Supplementary Table 1.

### Cell viability assay

Cell viability was determined using the Cell Counting Kit 8 (CCK8) assay. Briefly, AMs or HELF cells were seeded into 96-well plates at a density of 104 cells per well and incubated for 24 h. Subsequently, 10 µL of CCK8 solution (Beyotime Biotechnology, Shanghai, China) was introduced to the wells at different time points and incubated at 37 °C for 4 h. Sample absorbance was measured at 450 nm using a spectrophotometer (Multiscan MK3, Thermo Fisher Scientific, USA).

## Enzyme-linked immunosorbent assay (ELISA) and iron assay

The PD-L2 levels in patient serum samples and AM supernatants were determined by the Human PD-L2 SimpleStep ELISA kit (ab231928, Abcam, Cambridge, UK). The iron levels in the HELF cells were measured using the Iron Assay kit (ab83366, Abcam, Cambridge, UK). The assays were performed following the manufacturer’s instructions and the sample absorbance was measured at 450 nm and 593 nm, respectively, using a spectrophotometer (Multiscan MK3, Thermo Fisher Scientific, USA).

## Flow cytometry

The ratio of M1/M2 macrophages was determined using flow cytometry. Briefly, the cells were incubated with CD86-APC (381004, Biolegend, USA) and CD206-FITC antibodies (321104, Biolegend, USA) for 30 min. The numbers of APC- and FITC-positive cells were measured by a FACScan flow cytometer (BD Biosciences, USA). HELF cell apoptosis was determined by flow cytometry using the Annexin-V/PI Apoptosis Assay Kit (eBioscience, Thermo Fisher Scientific, USA) following the manufacturer’s instructions.

### Animal experiments

Eight-week-old wild-type (WT) and PD-L2 knockout (KO) mice were established on a C57BL/6 background (GemPharmatech Laboratory Animal Center, Jiangsu, China). The mice were housed in a specific pathogen-free environment at 25 °C with a 12 h light/12 h dark cycle. The animals had free access to water and food. All animal experiments were performed following the Laboratory Animal Guidelines and approved by the Soochow University Animal Care and Use Committee (Approval No. GPTAP002).

The CTD-ILD mice models were created by bleomycin (BLM, Yeasen Biotechnology, Shanghai, China) treatment. Baseline body weights were comparable between WT and KO mice (WT vs. KO: 24.24 ± 0.84 g vs. 24.46 ± 0.87 g; *P* = 0.572). The WT or KO mice were randomly divided into the treatment and control groups, resulting in four groups: WT-control group (*n* = 5), KO-control group (*n* = 5), WT-BLM group (*n* = 5), and KO-BLM group (*n* = 5). In the BLM treatment groups, BLM was administered via intratracheal injection (5 mg/kg of BLM in 50 µL of saline), whereas in the control groups, 50 µL of saline was intratracheally injected to each mouse. Mice were sacrificed 21 days after bleomycin administration for subsequent tissue collection and analysis.

### Immunofluorescence

After treatment, the cells or tissues were fixed and incubated with primary antibodies against PD-L2 (1:100, ab288298, Abcam, Cambridge, UK) or CD86 (1:100, ab280081, Abcam, Cambridge, UK) at 4 °C overnight. Subsequently, the cells were incubated with the corresponding secondary antibodies for 1 h in dark. The cells were then stained with DAPI (Sigma, USA), and imaged using a fluorescence microscope (Zeiss, Germany).

### Statistical analysis

Data were analyzed using the GraphPad Prism software 8.0 (San Diego, USA) and SPSS 22.0 software. For cell experiments, data are presented as the mean ± standard deviation (SD) of three independent replicates. For animal experiments, data are presented as the mean ± SD of five mice. Data normality was determined using the Shapiro-Wilk method. Homogeneity of variance test was performed using Levene’s method. Normally distributed data were compared using Student’s *t*-test (two-group comparison) or one-way ANOVA combined with post hoc Tukey’s multiple comparisons test (multi-group comparison). For non-normally distributed data involving more than two groups, comparisons were performed using the Kruskal–Wallis test followed by Dunn’s multiple comparisons test. Non-normally distributed data or data with inhomogeneous variance were compared using Mann-Whitney U test. P-values below 0.05 were considered statistically significant.

## Results

### Serum PD-L2 levels were low in CTD-ILD patients

**Fig. 1:**
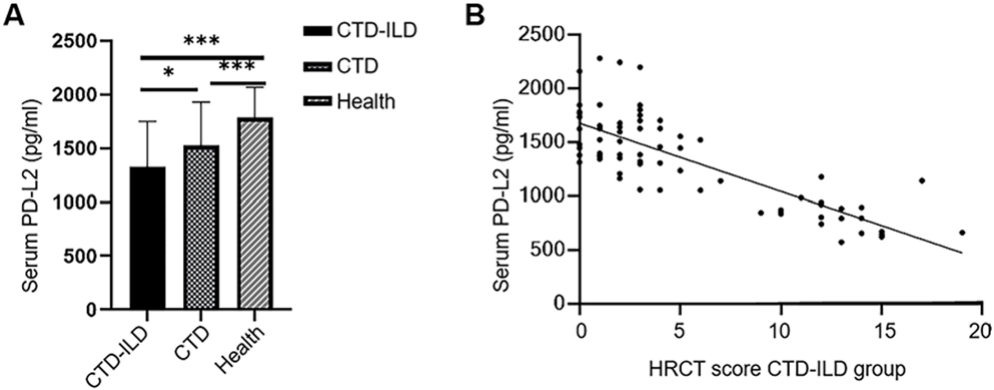
Serum PD-L2 levels were low in CTD-ILD patients. (**A**) Serum PD-L2 levels in CTD-ILD patients, CTD-nonILD patients, and healthy controls. Kruskal–Wallis test showed a significant difference among groups (H = 37.07, *P* < 0.0001), followed by Dunn’s multiple comparisons test indicating significantly lower PD-L2 levels in CTD-ILD patients compared with CTD-nonILD patients (adjusted *P* = 0.021) and healthy controls (adjusted *P* < 0.0001), as well as lower PD-L2 levels in CTD-nonILD patients than in healthy controls (adjusted *P* = 0.0009). (**B**) Pearson correlation analysis between serum PD-L2 levels and HRCT scores in CTD-ILD patients (*r* = − 0.80, *P* < 0.0001). *P < 0.05, **P < 0.01, ***P < 0.001

We first measured the serum PD-L2 levels in CTD-ILD patients. A total of 72 CTD-ILD patients, 76 CTD-nonILD patients, and 60 healthy controls were included. The baseline characteristics of these patients are shown in Supplementary Table 2. The serum PD-L2 concentrations differed significantly among the CTD-ILD, CTD-nonILD, and healthy control groups (Kruskal–Wallis test, H = 37.07, *P* < 0.0001). Post hoc Dunn’s multiple comparison further showed that the serum PD-L2 concentrations were significantly lower in CTD-ILD patients than in CTD-nonILD patients (adjusted *P* = 0.021) and healthy controls (adjusted *P* < 0.0001); additionally, PD-L2 levels in CTD-nonILD patients were also significantly lower than those in healthy controls (adjusted *P* = 0.0009) (Fig. 1A). Furthermore, we found that the serum PD-L2 levels were negatively correlated with the HRCT scores in the CTD-ILD patients (Pearson’s *r* = − 0.80, *P* < 0.0001) (Fig. 1B). These results suggest that the serum PD-L2 levels may be associated with the severity of CTD-ILD.

### Establishment of PD-L2 knockdown AMs

**Fig. 2:**
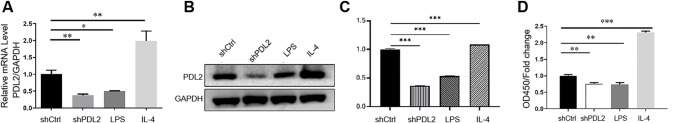
Establishment of PD-L2 knockdown alveolar macrophages. (**A**) RT-PCR. (**B**) Western blot. (**C**) Quantitative analysis of the Western blot results. (**D**) PD-L2 levels in the supernatant. *P < 0.05, **P < 0.01, ***P < 0.001

We then investigated the effect of PD-L2 knockdown on AM activation. Compared with the shCtrl group, the PD-L2 expression was significantly reduced in the shPD-L2 and LPS-treated groups, but was significantly enhanced in the IL-4-treated group, as confirmed by RT-PCR and Western blotting, respectively (all *P* < 0.05) (Fig. 2A-C). Consistent with these findings, PD-L2 levels in the macrophage supernatant (Fig. 2D) also followed a similar pattern: the PD-L2 levels were significantly decreased in the shPD-L2 and LPS-treated groups and elevated in the IL-4-treated group compared to the control (all *P* < 0.05).

### Effect of PD-L2 knockdown on cell viability and M1/M2 polarization

**Fig. 3:**
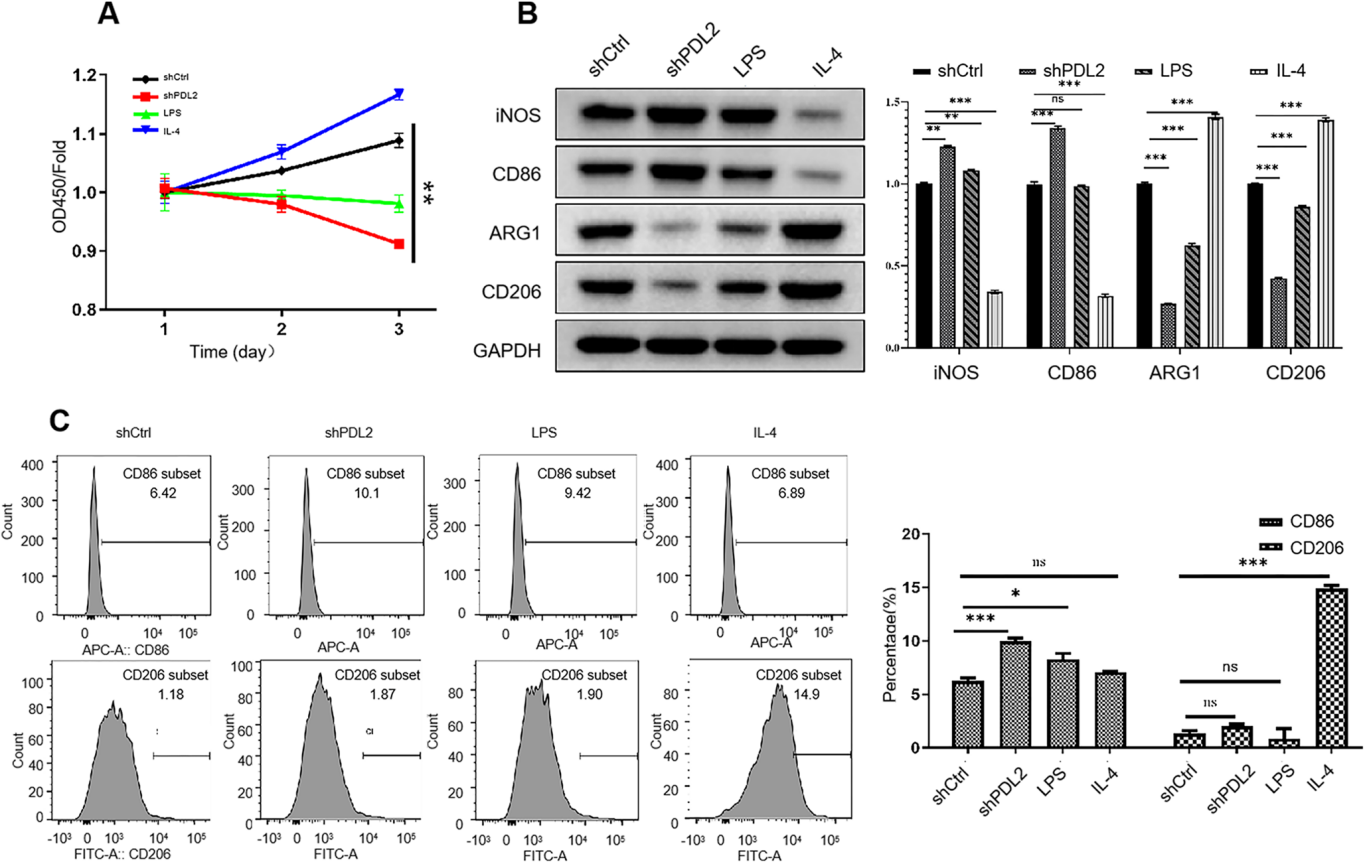
Effect of PD-L2 on AM cell viability and M1/M2 polarization of macrophage. (**A**) Cell viability. (**B**) Western blot. (**C**) Flow cytometry. *P < 0.05, **P < 0.01, ***P < 0.001

We then investigated the effects of PD-L2 knockdown on AM viability and polarization. The CCK-8 assay showed that PD-L2 knockdown significantly suppressed AM viability compared to the shCtrl transfection and LPS and IL-4 treatments (Fig. 3A). Next, we examined the expression levels of M1 and M2 macrophage markers, including iNOS, CD86, Arg1, and CD206. The expression levels of all four proteins were significantly different among the four treatments (one-way ANOVA, iNOS: F = 7354.09, *P* < 0.0001; CD86: F = 6878.69, *P* < 0.0001; Arg1: F = 6563.53, *P* < 0.0001; CD206: F = 10085.65, *P* < 0.0001). Post hoc Tukey’s multiple comparisons test showed that iNOS, Arg1, and CD206 differed significantly across all pairwise group comparisons (all *P* < 0.0001), whereas for CD86, no significant difference was observed between the shCtrl and LPS-treated groups (*P* = 0.26). Notably, compared to the shCtrl group, iNOS and CD86 were upregulated in PD-L2 knockdown AMs, whereas Arg1 and CD206 were downregulated (Fig. 3B). Flow cytometry analysis revealed that, compared to the shCtrl group, the ratio of CD86^+^ cells significantly increased in PD-L2 knockdown AMs (*P* < 0.05), although no significant change was observed in CD206^+^ cells (Fig. 3C). These results suggest that PD-L2 knockdown may promote the M1 polarization.

### PD-L2 knockdown AMs regulated apoptosis, fibrosis, and ferroptosis in lung fibroblasts

**Fig. 4:**
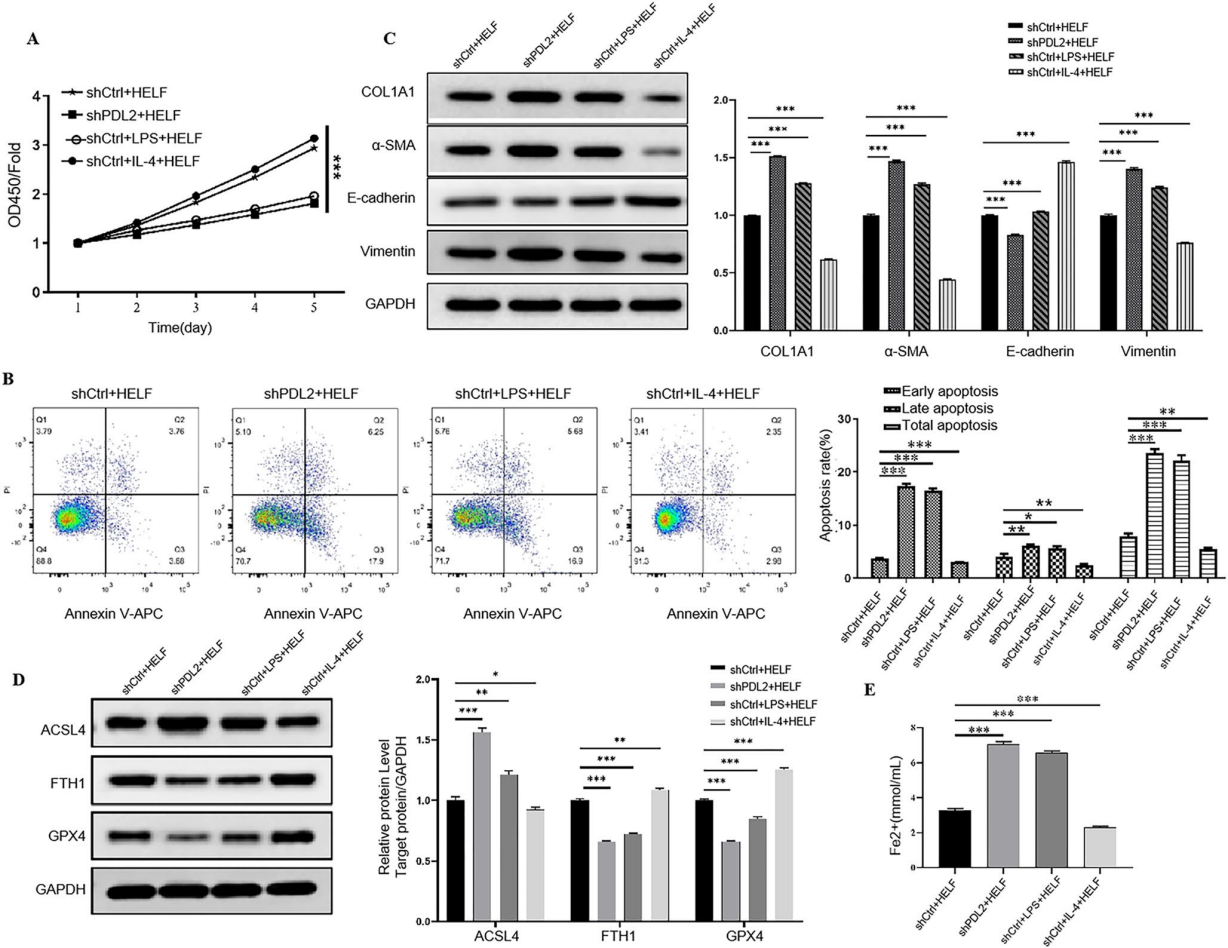
PD-L2 knockdown AMs regulated apoptosis, fibrosis, and ferroptosis in lung fibroblasts. (**A**) Cell viability. (**B**) Cell apoptosis. (**C**) Western blot. (**D**) Western blot. (**E**) Iron contents. *P < 0.05, **P < 0.01, ***P < 0.001

We then sought to study the effects of PD-L2 knockdown AMs on lung fibroblast cells. The HELF cells were first treated with TGF-β to promote fibrosis and co-cultured with the AMs. CCK-8 assay clearly showed that PD-L2 knockdown AMs and LPS-treated AMs significantly reduced the viability of HELF cells (Fig. 4A). Furthermore, flow cytometry results demonstrated that, compared to shCtrl transfection and IL-4 treatment, PD-L2 knockdown AMs and LPS-treated AMs significantly promoted HELF cell apoptosis (Fig. 4B). We then examined the expression of fibrosis-related markers, and Western blot analysis showed that HELF cells exposed to PD-L2 knockdown AMs or LPS-treated AMs exhibited increased expression of COL1A1, α-SMA, and vimentin, accompanied by reduced E-cadherin expression, compared with the control group (Fig. 4C).

We also explored whether PD-L2 knockdown AMs could induce ferroptosis in HELF cells. Western blotting showed elevated ACSL4 but reduced GPX4 and FTH1 levels in HELF cells co-cultured with PD-L2 knockdown AMs or LPS-treated AMs compared to the shCtrl group (all *p* < 0.05) (Fig. 4D). Furthermore, the cellular iron levels were also significantly enhanced in these two groups compared to the shCtrl and IL-4-treated groups (all *p* < 0.001) (Fig. 4E). Moreover, we explored the effects of AM supernatants on the HELF cells. Within expect, the AM supernatants showed similar effects compared to co-culture (Fig. S1A-E), in cell viability, apoptosis, fibrosis related markers, and ferroptosis. These results collectively suggest that PD-L2 knockdown in AMs could induce apoptosis, fibrosis, and ferroptosis in HELF cells, similar to the effect exhibited by LPS-stimulated M1 macrophages.

#### PD-L2 knockout regulates macrophage polarization in vivo

**Fig. 5:**
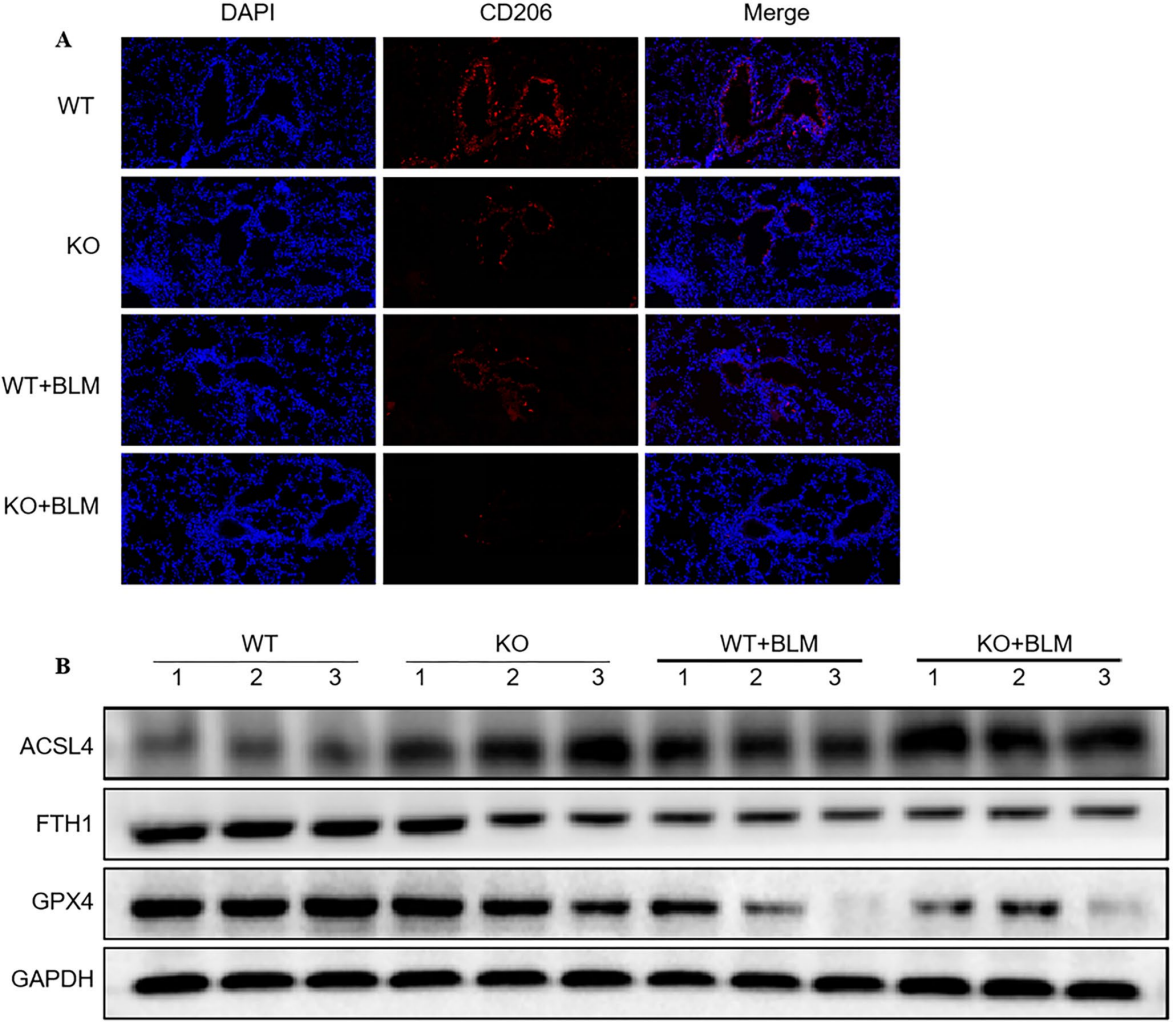
PD-L2 knockout regulates macrophage polarization in vivo. (**A**) Representative immunofluorescence images. DAPI (blue) was used for nuclear staining. Scale bar, 100 μm. (**B**) Western blot

Finally, we investigated the effects of PD-L2 knockout on macrophage polarization and lung tissue ferroptosis in vivo. Immunofluorescence showed CD206^+^ macrophages in the WT mouse lung tissue (Fig. 5A), the number of which was significantly reduced in PD-L2 knockout mice, BLM-treated WT mice, and BLM-treated PD-L2 knockout mice, suggesting that both PD-L2 deficiency and BLM exposure impaired M2 macrophage polarization in vivo. Western blotting further showed that PD-L2 knockout resulted in increased ACSL4 levels and reduced GPX4 and FTH1 levels. BLM further elevated ACSL4 levels and reduced GPX4 and FTH1 levels in both WT and PD-L2 knockout mice (Fig. 5B). These findings suggest that PD-L2 deficiency impairs M2-skewed macrophage polarization and exacerbates ferroptosis in vivo.

## Discussion

In this study, we identified PD-L2 as a critical regulator of macrophage function and fibroblast fate in the context of CTD-ILD both in vitro and in vivo. These findings highlight the importance of macrophage PD-L2 in regulating both inflammatory and fibrotic responses in CTD-ILD and suggest a novel link between immune regulation and fibroblast cell death.

Previous studies have shown that PD-L2 expression is closely associated with macrophage M2 polarization [20, 21]. In addition, infection with *Capillaria hepatica*, a zoonotic parasite, could promote PD-L2 expression in M2 macrophages that regulate Th2-biased regulatory immune responses and facilitate the survival of parasitic worms or eggs within the infected liver [22]. This study extends these observations by demonstrating that the knockdown of PD-L2 in macrophages can promote M1 polarization and activate the apoptosis, ferroptosis, and regulate expression of fibrosis related markers. In the context of CTD-ILD, it is well-established that M1 macrophage infiltration is strongly associated with tissue damage and disease severity [23], whereas M2 macrophages dominate in anti-inflammatory or tissue repair processes [24]. These insights suggest that enhancing PD-L2 expression or signaling in macrophages may represent a potential therapeutic strategy for CTD-ILD. Moreover, the significant correlation between serum PD-L2 levels and disease severity suggests that PD-L2 may serve as a potential biomarker for early diagnosis and disease monitoring in CTD-ILD. In addition, we observed that serum PD-L2 levels in CTD-nonILD patients were also significantly lower than those in healthy controls, suggesting that reduced PD-L2 expression may be a common feature of CTDs even in the absence of the onset of interstitial lung involvement. This finding further supports the potential of PD-L2 as an early immunological indicator in CTD-related conditions.

Interestingly, a recent study in post-COVID-19 patients reported that elevated serum PD-L2 levels were associated with persistent lung lesions and altered CD4/CD8 ratios at one year following infection [25]. While this appears to contradict our finding that serum PD-L2 levels are reduced in CTD-ILD patients with more severe disease, the discrepancy likely reflects fundamental differences in disease pathogenesis. In chronic infection models, including post-viral and parasitic infections [22, 26], elevated PD-L2 levels may represent prolonged immune dysregulation or compensatory anti-inflammatory signaling, often accompanied by enhanced M2-like macrophage activity and fibrotic remodeling. In contrast, CTD-ILD is driven by chronic autoimmune activation, where disrupted immune regulation may lead to reduced PD-L2 expression, increased M1 macrophage polarization, and immune-mediated tissue injury. These observations collectively highlight the context-dependent role of PD-L2 and underscore the importance of disease-specific interpretation of its clinical significance, which requires further investigation to fully elucidate the differential roles of PD-L2 in infection-related and autoimmune-related lung injury.

Although PD-L1 and PD-L2 are both ligands of PD-1 and belong to the B7 family, accumulating evidence indicates that they play non-redundant roles in immune regulation. PD-L1 is broadly expressed across immune and non-immune cells and is primarily induced by inflammatory cues such as IFN-γ [27–29], serving as a dominant immune checkpoint that restrains excessive immune activation. Moreover, current evidence does not support a consistent or direct association between PD-L1 expression and macrophage polarization states [30]. In contrast, PD-L2 exhibits a more restricted expression pattern, largely confined to antigen-presenting cells, including macrophages and dendritic cells [31, 32], and is more selectively regulated during macrophage activation and polarization. Emerging studies suggest that PD-L2 expression is closely associated with macrophage functional states, particularly M2-like programs [20, 21]. This divergence suggests that PD-L2, but not PD-L1, may play unique roles in macrophage behavior in specific disease contexts. Therefore, focusing on PD-L2 may provide a more refined understanding of macrophage polarization and its downstream impact on fibroblast responses, particularly in the context of CTD-ILD.

An interesting finding of our study is that PD-L2-deficient macrophages induce both fibrotic activation and ferroptosis in HELF cells, not only through direct co-culture but also via macrophage-conditioned supernatants. Specifically, fibroblast activation was characterized by increased expression of profibrotic markers, including COL1A1, α-SMA, and vimentin, accompanied by a reduction in E-cadherin expression, a hallmark of epithelial-mesenchymal transition. This observation suggests that soluble factors, such as exosomes, secreted by macrophages, may mediate fibroblast dysfunction in the absence of direct cell-cell contact. While previous studies have emphasized the role of macrophages in promoting fibrosis through pro-fibrotic cytokines such as TGF-β and IL-1β [33, 34], the ability of macrophages to induce ferroptosis in fibroblasts has been less well characterized. Given the critical role of ferroptosis in lung fibrosis [35], our data provide new evidence that macrophages with PD-L2 knockdown, likely exhibiting an M1-skewed phenotype, may release pro-ferroptotic signals that sensitize fibroblasts to lipid peroxidation and iron accumulation. These findings expand the current understanding of macrophage-fibroblast crosstalk and point to a dual role for PD-L2-deficient macrophages in exacerbating both cell death and fibrotic remodeling.

Despite the strengths of our study, several limitations should be acknowledged. Firstly, while we demonstrated that knockdown of PD-L2 in macrophages leads to phenotypic changes and functional alterations, the downstream intracellular signaling pathways triggered by PD-L2 deficiency remain poorly understood. Further investigation is needed to clarify how loss of PD-L2 alters transcriptional and metabolic programs in macrophages. Secondly, although we observed significant effects of PD-L2-deficient macrophages on fibroblasts in vitro, the in vivo relevance of these findings requires further validation. In particular, using a macrophage-specific PD-L2 knockout animal model would help isolate the contribution of macrophage-derived PD-L2 in the pathogenesis of CTD-ILD. Thirdly, while conditioned medium from PD-L2-deficient macrophages was sufficient to induce fibroblast apoptosis and fibrosis, we did not perform an in-depth analysis of the soluble mediators responsible for these effects. Identifying the specific cytokines, exosomes, or metabolites involved will be essential for understanding the molecular basis of macrophage-fibroblast communication in fibrotic lung disease. Fourthly, we did not collect data on prior COVID-19 infection status during patient enrollment. As recent studies suggest that serum PD-L2 levels may be altered in post-COVID lung disease [25], the lack of this information represents a potential confounding factor and may limit the interpretation of our findings.

In summary, our findings highlight the critical role of macrophage PD-L2 in regulating fibroblast responses and fibrotic progression in CTD-ILD, providing new insights into the cellular crosstalk involved in tissue remodeling in autoimmune lung disease. These results suggest that PD-L2 may be associated with disease severity and represent a pathway of interest for modulating macrophage activation and fibrotic responses in CTD-ILD, warranting further investigation in preclinical models.

## Supplementary Information

Below is the link to the electronic supplementary material.

Supplementary Material 1

Supplementary Material 2

## Data Availability

The datasets used and/or analyzed during the current study are available from the corresponding author on reasonable request.

## References

[1] Spagnolo P, Distler O, Ryerson CJ, Tzouvelekis A, Lee JS, Bonella F, et al. Mechanisms of progressive fibrosis in connective tissue disease (CTD)-associated interstitial lung diseases (ILDs). Ann Rheum Dis. 2021;80(2):143–50. 33037004 10.1136/annrheumdis-2020-217230 PMC7815631

[2] Enomoto N. Relationship between idiopathic interstitial pneumonias (IIPs) and connective tissue disease-related interstitial lung disease (CTD-ILD): A narrative review. Respiratory Invest. 2024;62(3):465–80. 10.1016/j.resinv.2024.03.006 38564878

[3] Vij R, Strek ME. Diagnosis and treatment of connective tissue disease-associated interstitial lung disease. Chest. 2013;143(3):814–24. 23460159 10.1378/chest.12-0741 PMC3590889

[4] Althobiani MA, Russell AM, Jacob J, Ranjan Y, Folarin AA, Hurst JR, et al. Interstitial lung disease: a review of classification, etiology, epidemiology, clinical diagnosis, pharmacological and non-pharmacological treatment. Front Med. 2024;11:1296890. 10.3389/fmed.2024.1296890 PMC11063378 38698783

[5] Maher TM. Interstitial Lung Disease: A Review. JAMA. 2024;331(19):1655–65. 38648021 10.1001/jama.2024.3669

[6] Cerro Chiang G, Parimon T. Understanding Interstitial Lung Diseases Associated with Connective Tissue Disease (CTD-ILD): genetics, cellular pathophysiology, and biologic drivers. Int J Mol Sci. 2023;24(3):2405. 10.3390/ijms24032405 PMC9917355 36768729

[7] Tseng CC, Sung YW, Chen KY, Wang PY, Yen CY, Sung WY et al. The role of macrophages in connective tissue disease-associated interstitial lung disease: focusing on molecular mechanisms and potential treatment strategies. Int J Mol Sci. 2023;24(15):11995. 10.3390/ijms241511995 PMC10419312 37569370

[8] Brady NJ, Chuntova P, Schwertfeger KL, Macrophages. Regulators of the Inflammatory Microenvironment during Mammary Gland Development and Breast Cancer. Mediat inflamm. 2016;2016:4549676. 10.1155/2016/4549676 PMC4739263 26884646

[9] Yunna C, Mengru H, Lei W, Weidong C. Macrophage M1/M2 polarization. Eur J Pharmacol. 2020;877:173090. 32234529 10.1016/j.ejphar.2020.173090

[10] Gu Y, Lawrence T, Mohamed R, Liang Y, Yahaya BH. The emerging roles of interstitial macrophages in pulmonary fibrosis: A perspective from scRNA-seq analyses. Front Immunol. 2022;13:923235. 36211428 10.3389/fimmu.2022.923235 PMC9536737

[11] Jiang Y, Cai R, Huang Y, Zhu L, Xiao L, Wang C, et al. Macrophages in organ fibrosis: from pathogenesis to therapeutic targets. Cell death discovery. 2024;10(1):487. 39632841 10.1038/s41420-024-02247-1 PMC11618518

[12] Lee JH, Jang JH, Lee S, Her M. Immunopathogenic mechanisms in connective tissue disease-associated interstitial lung disease: incessant loop of immunity to fibrosis. Int J Mol Sci. 2025;26(24):12126. 10.3390/ijms262412126 PMC12732998 41465549

[13] Evans RA, Tian YC, Steadman R, Phillips AO. TGF-beta1-mediated fibroblast-myofibroblast terminal differentiation-the role of Smad proteins. Exp Cell Res. 2003;282(2):90–100. 12531695 10.1016/s0014-4827(02)00015-0

[14] Zhou BW, Liu HM, Xu F, Jia XH. The role of macrophage polarization and cellular crosstalk in the pulmonary fibrotic microenvironment: a review. Cell communication signaling: CCS. 2024;22(1):172. 38461312 10.1186/s12964-024-01557-2 PMC10924385

[15] Zhu L, Fu X, Chen X, Han X, Dong P. M2 macrophages induce EMT through the TGF-β/Smad2 signaling pathway. Cell Biol Int. 2017;41(9):960–8. 28493530 10.1002/cbin.10788

[16] Wang Y, Du J, Gao Z, Sun H, Mei M, Wang Y, et al. Evolving landscape of PD-L2: bring new light to checkpoint immunotherapy. Br J Cancer. 2023;128(7):1196–207. 36522474 10.1038/s41416-022-02084-y PMC10050415

[17] Ellis JS, Guloglu FB, Tartar DM, Hoeman CM, Haymaker CL, Cascio JA, et al. APCs expressing high levels of programmed death ligand 2 sustain the development of CD4 T cell memory. J Immunol (Baltimore Md : 1950). 2010;185(6):3149–57. 10.4049/jimmunol.1000810 PMC3057906 20709947

[18] Rodríguez-García M, Porichis F, de Jong OG, Levi K, Diefenbach TJ, Lifson JD, et al. Expression of PD-L1 and PD-L2 on human macrophages is up-regulated by HIV-1 and differentially modulated by IL-10. J Leukoc Biol. 2011;89(4):507–15. 21097698 10.1189/jlb.0610327 PMC3058820

[19] Loke P, Allison JP. PD-L1 and PD-L2 are differentially regulated by Th1 and Th2 cells. Proc Natl Acad Sci USA. 2003;100(9):5336–41. 12697896 10.1073/pnas.0931259100 PMC154346

[20] Li Y, Tan Y, Li X, Chen X, Wang L, Zhang L, et al. Loss of LXN promotes macrophage M2 polarization and PD-L2 expression contributing cancer immune-escape in mice. Cell death discovery. 2022;8(1):440. 36323670 10.1038/s41420-022-01227-7 PMC9630456

[21] Yang H, Zhang Q, Xu M, Wang L, Chen X, Feng Y, et al. CCL2-CCR2 axis recruits tumor associated macrophages to induce immune evasion through PD-1 signaling in esophageal carcinogenesis. Mol Cancer. 2020;19(1):41. 32103760 10.1186/s12943-020-01165-x PMC7045401

[22] Huang M, Li X, Zheng X, Wang F, Zou Y, Wang L. PD-L2 blockade exacerbates liver lesion in mice infected with capillaria hepatica through reducing alternatively activated macrophages. Trop Med Infect disease. 2023;8(1):46. 10.3390/tropicalmed8010046 PMC9866821 36668953

[23] Chen S, Chen L, Ye L, Jiang Y, Li Q, Zhang H et al. PP2A-mTOR-p70S6K/4E-BP1 axis regulates M1 polarization of pulmonary macrophages and promotes ambient particulate matter induced mouse lung injury. J Hazard Mater. 2022;424(Pt C):127624. 10.1016/j.jhazmat.2021.127624 34740159

[24] Rao LZ, Wang Y, Zhang L, Wu G, Zhang L, Wang FX, et al. IL-24 deficiency protects mice against bleomycin-induced pulmonary fibrosis by repressing IL-4-induced M2 program in macrophages. Cell Death Differ. 2021;28(4):1270–83. 33144678 10.1038/s41418-020-00650-6 PMC8027679

[25] Buendia-Roldan I, Martínez-Espinosa K, Aguirre MJ, Aguilar-Duran H, Palma-Lopez A, Palacios Y, et al. Persistence of lung structural and functional alterations at one year post-COVID-19 is associated with increased serum PD-L2 levels and altered CD4/CD8 ratio. Immun Inflamm Dis. 2024;12(7):e1305. 39031504 10.1002/iid3.1305 PMC11259001

[26] Saha B, Kodys K, Szabo G, Hepatitis C, Virus-Induced. Monocyte Differentiation Into Polarized M2 Macrophages Promotes Stellate Cell Activation via TGF-β. Cell Mol Gastroenterol Hepatol. 2016;2(3):302–e168. 28090562 10.1016/j.jcmgh.2015.12.005 PMC5042356

[27] Hu L, Sun C, Yuan K, Yang P. Expression, regulation, and function of PD-L1 on non-tumor cells in the tumor microenvironment. Drug Discovery Today. 2024;29(11):104181. 39278561 10.1016/j.drudis.2024.104181

[28] Chen RY, Zhu Y, Shen YY, Xu QY, Tang HY, Cui NX, et al. The role of PD-1 signaling in health and immune-related diseases. Front Immunol. 2023;14:1163633. 37261359 10.3389/fimmu.2023.1163633 PMC10228652

[29] Abiko K, Matsumura N, Hamanishi J, Horikawa N, Murakami R, Yamaguchi K, et al. IFN-γ from lymphocytes induces PD-L1 expression and promotes progression of ovarian cancer. Br J Cancer. 2015;112(9):1501–9. 25867264 10.1038/bjc.2015.101 PMC4453666

[30] Cai H, Zhang Y, Wang J, Gu J. Defects in Macrophage Reprogramming in Cancer Therapy: The Negative Impact of PD-L1/PD-1. Front Immunol. 2021;12:690869. 34248982 10.3389/fimmu.2021.690869 PMC8260839

[31] Loke Pn, Allison JP. PD-L1 and PD-L2 are differentially regulated by Th1 and Th2 cells. Proceedings of the National Academy of Sciences. 2003; 100(9):5336-41. 10.1073/pnas.0931259100 PMC154346 12697896

[32] Dail M, Yang L, Green C, Ma C, Robert A, Kadel EE, et al. Distinct Patterns of PD-L1 and PD-L2 Expression By Tumor and Non-Tumor Cells in Patients with MM, MDS and AML. Blood. 2016;128(22):1340.

[33] Murray LA, Chen Q, Kramer MS, Hesson DP, Argentieri RL, Peng X, et al. TGF-beta driven lung fibrosis is macrophage dependent and blocked by Serum amyloid P. Int J Biochem Cell Biol. 2011;43(1):154–62. 21044893 10.1016/j.biocel.2010.10.013

[34] Kolb M, Margetts PJ, Anthony DC, Pitossi F, Gauldie J. Transient expression of IL-1beta induces acute lung injury and chronic repair leading to pulmonary fibrosis. J Clin Investig. 2001;107(12):1529–36. 11413160 10.1172/JCI12568 PMC200196

[35] Hu Y, Huang Y, Zong L, Lin J, Liu X, Ning S. Emerging roles of ferroptosis in pulmonary fibrosis: current perspectives, opportunities and challenges. Cell death discovery. 2024;10(1):301. 38914560 10.1038/s41420-024-02078-0 PMC11196712

