# Supplementary figures and images for: PD-L2 deficiency in Alveolar macrophages drives fibrosis, apoptosis, and ferroptosis via M1 polarization in connective tissue disease-associated interstitial lung disease
Source: Clin Exp Med. 2026 Mar 13;26(1):193. doi: 10.1007/s10238-026-02115-5 (PMC13013153; doi:10.1007/s10238-026-02115-5)

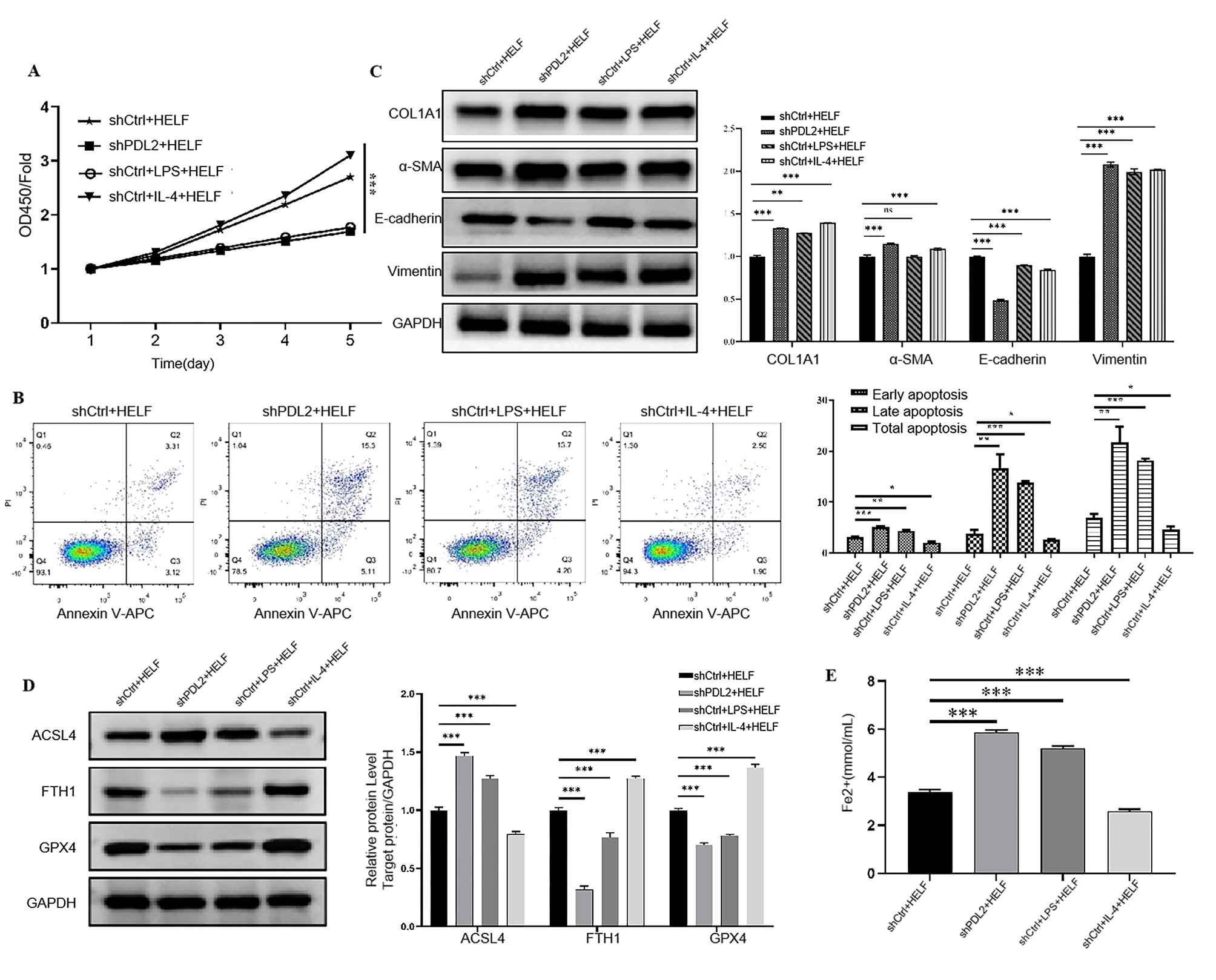

Supplement: Supplementary file 1 — Supplementary Material 1 [file 10238_2026_2115_MOESM1_ESM.tif]
