# Supplementary material for: PD-L2 deficiency in Alveolar macrophages drives fibrosis, apoptosis, and ferroptosis via M1 polarization in connective tissue disease-associated interstitial lung disease
Source: Clin Exp Med. 2026 Mar 13;26(1):193. doi: 10.1007/s10238-026-02115-5 (PMC13013153; doi:10.1007/s10238-026-02115-5)
Supplement: Supplementary file 2 — Supplementary Material 2 [file 10238_2026_2115_MOESM2_ESM.docx]

**Supplementary Table 1. List of antibodies used in this study.**

| Gene name | LOT | Manufacturer | Dilution ratios |
| --- | --- | --- | --- |
| PD-L2 | ab288298 | abcam | 1:1000 |
| iNOS | 95423S | UNIV | 1:1000 |
| CD86 | 91882S | UNIV | 1:2000 |
| ARG1 | 93668S | UNIV | 1:2000 |
| CD206 | 24595S | UNIV | 1:1000 |
| COL1A1 | ab138492 | abcam | 1:2000 |
| α-SMA | ab5831 | abcam | 1:2000 |
| E-cadherin | ab40772 | abcam | 1:2000 |
| Vimentin | ab92547 | abcam | 1:2000 |
| ACSL4 | ab155282 | abcam | 1:2000 |
| FTH1 | ab75972 | abcam | 1:2000 |
| GPX4 | ab125066 | abcam | 1:2000 |
| GAPDH | AF7021 | Affinity | 1:3000 |
| Goat Anti-Rabbit | AF7021 | Affinity | 1:3000 |

**Supplementary Table 2. Baseline characteristics of patients.**

| Group | n | Age (years)  *(mean*±*SD)* | Sex | | | Disease | | | | | | | | |
| --- | --- | --- | --- | --- | --- | --- | --- | --- | --- | --- | --- | --- | --- | --- |
|  |  |  | M | F | | RA | SS | SLE | | IIM | SSc | MCTD | Vasculitis | UCTD |
| CTD-ILD | 72 | 62.1±14.20 | 14 | | 58 | 11 | 25 | | 10 | 11 | 5 | 2 | 1 | 7 |
| CTD-nonILD | 76 | 51.36±15.02 | 10 | | 66 | 11 | 31 | | 16 | 1 | 4 | 3 | 3 | 7 |
| Healthy control | 60 | 54.62±14.92 | 10 | | 50 | - | - | | - | - | - | - | - | - |
| P |  | 0.0002 | 0.30 | | |  |  | |  |  |  |  |  |  |

M, male. F, female. RA, rheumatoid arthritis. SS, Sjögren’s syndrome. SLE, systemic lupus erythematosus. IIM, Idiopathic inflammatory myopathy. SSc, systemic sclerosis. MCTD, Mixed connective tissue disease. UCTD, undifferentiated connective tissue disease. CTD, connective tissue disease. ILD, interstitial lung disease.
